# Reply to Mr. John Mason Good's Remarks on Our Review of His Physiological System of Nosology

**Published:** 1817-12

**Authors:** 


*For the London Medical and Physical Journal.*

### *Reply to Mr. John Mason Good's Remarks on our Review of his Physiological System of Nosology.*

OUR readers will not suppose that we consider our reviews as oracular. They are intended to remind the author of what we conceive he may have overlooked, as well as to direct our readers in their choice of books, and the *grana salis* with which they should peruse some of them. At the same time, we wish never to be backward in acknowledging the information we derive, the new sentiments excited in our minds, and, where we have doubt, to request information from writers who, we conceive, before they venture to publish, have most maturely considered their subject. In all these respects, we have been particularly careful to acknowledge our obligations to Mr. Good; and, when he honoured us with his "Remarks," we waived the liberty he permitted us of "subjoining any *explanation*," not only because we thought it unfair, but because the author and his subject appeared to us worthy of a maturity of composition beyond an extemporaneous reply. We even afforded him an indulgence we dare not claim ourselves, that of transcribing a long passage from a former Journal. There are many reasons why we should gladly assume the same privilege; but we should think it injustice to our subscribers thus to fill our pages. We are the more anxious to explain this, in order that Mr. Good, should he honour us with a rejoinder, (which we promise to insert,) may not repeat such a request; and also that our readers may be careful to keep the whole controversy before them whenever they peruse a part of it. For this reason, we have, in what follows, directed to the pages in our Journal instead of transcribing them. We now proceed, 1st, to our defence; 2dly, to request further information from Mr. Good on some passages which he has left unnoticed; and, lastly, to restore a few sentences which we expunged in our manuscript or proofs before we were aware that the author would be so ready at explanation.

Mr. G. has used the words Nosology and Pathology "as nearly *parallel* terms, though not exactly synonymous." *Parallel*, when applied to words, can only mean such as may be *interchangeably* used, which is very much like synonymous. "They are directly synonymous in their etymology"—*Negatur:* because what Sauvages (*teste* Mr. Good) says of nosology, "*Morborum* SCIENTIA *seu habitus demonstrandi* QUIDQUID *de morbis affirmamus aut negamus*," is not a true definition of pathology, which relates only to the symptoms. The science of *medicine*, exclusive of surgery, has been usually divided into physiology, pathology, and therapeutics. Nosology, it is true, is part of the first, and might comprehend the other two. All this we admit, and that the inventors of this new science, as we consider it, by adding *methodica*, imply that only their *οδος*, or *way*, is different, as Celsus long since remarked of another set of philosophers (quasi *viam* quendam quam *μεθωδον* Græci nominant). Now, it is to this *οδος*, or march, as the French would call it, that we object, considering that the student is rather bewildered than assisted by its intricacies, and still more by the discordant opinions of his various directors. Mr. Good seems to us perfectly aware of this, when, in his title (PHYSIOLOGICAL SYSTEM) he not only drops the *οδος*, but seems to admit that the various *methods* were not formed on the principles of *physiology*, yet this is the science on which the regular *physician* founds his distinction from the empiric. But, though the title-page promises a "Physiological System," the "preliminary dissertation" teaches us to expect nothing more than an arrangement similar to those formed by naturalists, who are always careful to produce the most *perfect specimens*. Even in botany, too, we are told (p. 216) that a "*natural* system" is rather a theoretical than a practical idea; that therefore there seems very little probability it can ever be realized in medicine." Despairing, therefore, we presume, of accomplishing this part of his design, Mr. Good illustrates his plan by "*technology*," showing that, as the practical machinist learns to produce the same effect with fewer powers, so the nosologist is by practice enabled to explain himself in fewer words. Of this, which he calls *simplicity*, he gives us a specimen (p. 218) in his long article concerning Mariscæ, of which we shall take notice hereafter. This part of the "Remarks" is concluded thus:—" The mistake of the Reviewer consists in his confounding the one idea with the other, the *thing* with the *name*, the *science* with the *method* under which it is now taught.

taught. To suppose that there was no such *science* as nosology, the MORBORUM SCIENTIA, before this name was applied to it, is to suppose that there was no such thing as *morbid poisons* before Mr. J. Hunter used the term, or Dr. Adams (a still more favourite name with the Reviewer—see p.p. 229, 298, 312, 316, 319,) wrote a book upon the subject."

All that is contained in the first of these sentences having been already noticed, is only transcribed on account of its connexion with the second, which is still so illogically constructed that the *thing to be supposed* is first the *science* of diseases, and immediately after the *diseases* themselves. No inference, therefore, can be legitimate, and we can only allow Mr. Good the advantage of a happy juxta-position, and his *argumentum ad hominem*. We maintain it, then, that, though the science of medicine was not new when the word Nosology was introduced, and therefore that, if it means no more, such a word was unnecessary; yet that the science of *morbid poisons* was new when Mr. Hunter first undertook to point out the *οδος* of studying it, and felt the necessity of a new term to distinguish *morbid* from other animal poisons.

Before Mr. Good reminded us of it, we were aware how often the names of Hunter and Adams had occurred; and in some of the passages the reader will find their works mentioned without any name. The difference is, however, so trifling, that we allow Mr. Good the full force of his remark, and feel greatly obliged to him for this opportunity of repeating the circumstance by which we were led to select certain *genera* which led to the introduction of these names. *Odontia* was the first that presented itself according to the *physiological system*. The other genera were obtruded upon us by the blunder of the index-maker. Odontia we found, for the most part, a transcript of Mr. Hunter; and as, in our opinion, every deviation from the text was an error, and some of them very dangerous, could we do better than restore the text? In the order PHLOGOTICA—*Inflammations*, by Mr. G.'s suggestion, we referred the reader to Mr. Hunter's writings, in order to "elucidate the nature of arrangement introduced into the present method." P. 317. In *syphilis*, we were anxious to relieve our readers from *Frenk zchemti*, *nar farsi*, or *atesi farsi*, meaning ignis Persicus, a term "much older than any records we possess of syphilis,"—from *sanc huya* in the Hindu, *shuce* in Arabic, *merghi-mush* (mouse-bane in Persian,) in common language preparations of arsenic; from Hindu physicians or *Cabirajas*—from these to the the disease by its English name—to the well-known remedy and John Hunter. In the notes on *syphilódes*, we were told that the "keen and comprehensive mind" of Mr. John Hunter "first called the attention of practitioners to the idea of different poisons and different maladies." Afterwards Mr. Abernethy is said to have "put the question at rest;" and, at the end of the same paragraph, that to several appearances approaching to, but differing from, genuine syphilis, Mr. Abernethy gives the names of *pseudo-syphilitic diseases.*" In referring to Mr. Abernethy's early writings on this subject, we found him associating the names of Celsus, Hunter, and and Adams; and, in examining the latter, we found at least an attempt, not indeed at classification nor artificial arrangement, which Dr. Bateman and Mr. Good despair of, but at arrangement according to Celsus and Hunter (see p. 317). And does Mr. Good think much of a dozen lines thus occupied? Our remarks on Elephantiasis will occur hereafter.

We believe we were as much entertained as our readers by Mr. Good's witty remarks on our opinions concerning artificial arrangement in the productions of nature. When we read of "brilliant specimens" of "concordia discors," of "flaming bounds of time and space," *cum multis aliis*, it brought to our mind a set of people who talk incessantly in order to distract their hearers' attention from the object before him. The commonest reader must understand, that, in making an arrangement of natural productions beyond what may serve for a catalogue raisonnée, an uniformity in the appearance of the various specimens is expected much beyond any thing which we have detected in acute diseases. The attempt, in any branch of medicine, to say the least, appears premature; and that it is dangerous in acute diseases we have had so many concurrent proofs, as to render it unnecessary to say more on the subject.

So far from accusing our printer of inaccuracy (p. 363), we often find him correcting us, which no doubt he would have done in the proper names of Crichton and Sauvages, had they been as familiar to him as Sydenham, Mr. Hunter, and some others. We are, therefore, obliged to Mr. Good for this attention, and shall be still more obliged to him if he will have the goodness to mark any other errors, all which shall be noticed at the completion of our volume. Mr. Good is, however, much mistaken if he conceives us sharp in pointing out literal errors. We are too conscious of our frequent mistakes in a work which must appear by the end of the month, not to make every allowance for his nine years' labour. When, therefore, we met with ελεαφας, page 256, line 1 of his notes, we corrected it in the proof, (see Journal, p. 306,) without leaving the error and adding [ελεφας]. But, had we passed over so palpable an error as *ulcus* with a masculine adjective, we could scarcely expect to be treated with the tenderness we showed Mr. Good. In him we could only impute it, as we expressed ourselves, to *some unaccountable oversight* (p. 393). Mr. G. refers us to p. 248 for an explanation:—In our copy of his work, we can find no mention of a cancel. We presume, therefore, it was printed off before the error was detected: if so, it was unpardonable in the publisher to furnish a Reviewer with such a copy. Not that we could have satisfied ourselves with such an apology in our Journal, for, full as we are of literal blunders, we should have cancelled a page, or a sheet, a second or third time before we exposed such an eye-sore as *Ulcus vitiosus, callosus, cancrosus, sinuosus, fistulosus,* and *cariosus.*

When we spoke of Odontia *senilium* as an inelegance, we were in hopes Mr. Good would have understood us, and not obliged us to ask, what would he have thought of *salacitas puerilium* or *s. juvenilium?* and whether *s. senilium* is not as absurd, though we used a more gentle expression? We shall also be thankful for further information concerning *syphilódes.* But this relates to our request that Mr. Good will set us to rights in some other passages. And, first, in the specimen he gives us of *simplicity* (p. 219):—Marisca is preferred to the Gaza (γαζα), as a word better known. A great many learned remarks follow on hæmorrhoides and on Sauvages's mistake concerning the γυνη αιμορρουσα. We are told our English word *marsh* is derived from *marisca;* and that *gaza,* the term used by Aristotle, is by Hesychius and Scaliger called a Persian word, meaning *thesaurus tributus reditus,* a treasury,—that the term is rather Arabic than Persian, &c.—that the Arabic root is *khasi,* a blush, or ruddy flush, from whatever cause: hence the verb *(khasa)* to make one blush; and hence *khazan,* in Persian, signifies autumn, or the season of fulness or erubescence,—while *khasan,* in Arabic, is a garner, treasury, or conservatory, literally *cella, cellula, gaza,* or *gazophylacia,* as explained by Hesychius. In all this we submit to Mr. Good, only requesting to know what it all means, and taking the freedom to remark, that, after reading it, we were no longer surprised at the etymology by which some have proved that the Emperor Charlemagne was the son of a linen-draper, inasmuch as the name of his father, King *Pepin,* is only a corruption of the word *napkin*—napekin, nepekin, pepekin, pepin. Another instance, still more to the purpose, and not so entirely unconnected with medicine as blushing, autumn, treasury, and

King Pepin, is the manner in which the family of Milorbetti proved their relationship with our Thomas à Becket, in consequence of which they were allowed the privilege of having the relics of that saint brought into a sick chamber. A bishop, they urged, in England, is called My Lord; but the apposition of two such consonants as *d* and *b* is intolerable to an Italian ear. The gutteral sound of *k* is still worse; and the termination of *tti* is peculiarly grateful in proper names. Hence My Lord Becket becomes Milorbetti.

After this long digression, we shall no more object to Mr. Good's etymologies, especially as we acknowledge our ignorance of Persian, Arabic, and Hindu; but still we remain no better satisfied with his improved *simplicity* in considering fici, mariscæ, and hæmorrhoides as the same disease. We have since discovered, too, that, depending on Mr. Good's reference to Sauvages, we have done that author great injustice. In his first edition, he makes a proper distinction between fici and hæmorrhoides. The former, under genus xxiv., he calls condyloma ficus, *podicis* vel vulvæ. Mariscæ, on the contrary, in genus xxiv., he describes as in *ani* margine enati. He even adds, as if fearful of some future nosologist, "Cave ne confundas condylomata cum mariscis." Perhaps we shall be accused of having stolen the caution we gave at p. 325 from this passage, and we are so well satisfied with the support of such authority as to submit to the imputation. Sauvages very properly, too, corrects the errors of those who ascribe *fici* to syphilis, urging that they were well known to Martial. It should be remarked, however, that, notwithstanding the similarity of sound, and probably the same *radical*, yet the nouns for a *fruit* and for the *disease* were differently declined, and, some grammarians assert, of a different gender. There are, we admit, those who dispute the latter, and even doubt the authority of Martial. "In tertia notione (morbi) volunt grammaticorum filii esse masculini generis adducti ut videtur Epigrammate Martialis,

> Cum dixi ficus, rides quasi barbara verba,
> Et dici ficos Cœciliane jubes:
> Decemus ficus quas scimus in arbore nasci;
> Decemus ficos Cœciliane tuos.

Neque enum alibi apud bonæ notæ auctorem cum adjectivo vel masc. vel femineo junctum reperiri puto sed," &c. We must stop here, lest Juvenis, fresh from school, should recollect all that we have said in his "Propria quæ maribus," and in old Ainsworth or Morell. It will be safer to begin ourselves—

Incerti

Incerti generis sunt talpa et dama, canalis,

Pro morbo ficus fici dans atque phaselus.

*Propria quæ maribus, Nomina generis uncerti.*

Should our readers think proper to consult old Ainsworth, they will find our learned quotations and remarks under the word Ficus. They will, however, perceive how much longer we could have made them, had we *thought proper.* But to let them into the secret, we had not a spare copy to be at the mercy of scissors and paste, and we found transcribing extremely dull. In making this confession to our young friend, we have inadvertently let all our readers into the mystery of book-making. This was ill-judged; but, in this article, we have so perpetually expunged, that we are now determined to leave the passage as it is. It may, at least, serve to show Mr. Good, that how scanty soever he may think our *original* matter, (see page 363,) we could have *measured* out more even from our dictionary, without copying from his book, especially if we add the quantity of original matter we crossed through.

The part we are now about to restore was expunged, not only on account of the length of the article, but for a reason which may surprize Mr. Good and many of our readers. It was, because the name of Adams occurred so often. Thus, when we regretted that Mr. G. had, in his *Syphilodes,* attended so little to Mr. Hunter's nicer distinctions, and even passed over the term "morbid poisons," though adopted by Jenner and Abernethy, those were not the only names produced in our first manuscript; yet, when we came to the end of this article, (*Syphilodes,*) and found "Sibbens, Sivvens, (query,) the varieties," &c. (p.313,) we confess the great inclination we felt to remind Mr. Good of Dr. Adams's journey into Scotland, and his residence during the harvest in a part of the country in which Sivvens, by the account of all indigenous writers, prevails most, and at that particular season. As yaws too was unnoticed in *Syphilodes,* though at one time considered a *species* of syphilis, we could not restrain our curiosity from examining under what head it was arranged in Mr. Good's Physiological System. And, when we found the names of Sauvages, Sagar, Cullen, Parr, and Young, none of whom pretend to have seen yaws; of Winterbottom, Dancer, and Ludlam, to the last of "whom we are indebted, perhaps, for the best history which has been given of the disease," when we reflected that this history is contained in an inaugural dissertation, consequently written at an early period of life, and not easily procured, and that *Morbid Poisons* contained the substance of these three last writers, and also of

Drs. Hillary, Wright, and others; that, disregarding all apprehensions of contagion, which Dr. Ludlam remarks had heretofore prevented the surgeons of the West Indies from examining yaws with sufficient minuteness, the author had watched all the symptoms of a case which came under his own care before the commencement of the eruption, and had dissected the pustules in their several stages. When we reflected too, that his account of this case was so well received by the Medical Society of London as to procure the writer a silver medal for the best paper, though he was not at that time an officer in the Society, but at a distance from home; most of all, when we recollected our promise to Juvenis, we confess we found great difficulty in not referring to the book, the title of which Mr. Good has been kind enough to introduce in his "Remarks." The only recompense we could make was to refer with greater freedom to some others. Thus, the name of Celsus we cannot conceive occurs less than a dozen times, of Sydenham and Cullen at least half as often, of Aretæus nearly the same; Crichton somewhere properly spelt, as well as in the error Mr. G. has corrected; Hey, we believe, as often as the name of which we are reminded, and in some of the pages cited. Yet, excepting Aretæus, none of these wrote expressly on the *genera* we selected from Mr. Good's work.

Even this difficulty was trifling compared to our embarrassment under the article Elephantiasis. Here, to use the expression of a well-known author, "Phrygia and Pamphylia, Asia, Cappadocia, and Pontus," would have given us as much information as we met with in Mr. Good; and, unfortunately, every writer on whom we could depend, or whom we could understand, from Aretæus to the present, inclusive, was left unnoticed. What distressed us the more was, that, should we even attain the "oriental" languages, we must trust to dictionaries for a time, and that these, like the writings of Alsahavius, would afford us nothing but "perplexity" and "jumble." Could we, therefore, do otherwise than produce the records of those who *had seen the disease*? especially as all (with one exception) agree in the account of what they saw, and are not contradicted by Dr. Bateman, but only by the early authorities he produces.

After all, we pretend not to say, that the Elephantiasis, which occurred at St. Bartholomew's, and lately at Edinburgh, was the same disease as the *juzam* of the land of Uz. But then, Dr. Bateman and his learned friend differ as to the contagious property of juzam. And here, we cannot help thinking that Dr. B., however inferior his oriental attainments may be, must be right: first, because neither

Job's vixen of a wife, nor his good-natured comforters, ever reproach him with the contagious nature of his distemper, though they are not backward in telling him how disagreeable a figure he made; next, though they remained with him in silence seven days and seven nights, and had afterwards long conversations with him, yet neither Elephaz the Temanite, Bildad the Shuhite, nor Zophar the Naamanite, appear to have introduced the disease among their countrymen; lastly, we cannot help thinking, that so considerate a man as Job would have refused all intercourse with his friends, whilst afflicted with a contagious disease of so loathsome a description. We conceive, therefore, either that juzam was not Job's disease, or that the contagion of juzam, like that of Elephantiasis, is still undecided.

If we did not suppose our readers, by this time, satisfied, how easy it is to furnish matter without transcribing from the works we analyse; we could treat them with a long quotation from Mead, who, with Mr. Good, conceives Job's disease to be Elephantiasis. It is true, that, ignorant of Arabic, Mead could know nothing of juzam, and, in his time, Elephantiasis had not been detected in St. Bartholomew's Hospital. Still, as we have an old copy which we might have cut and pasted, our readers must give us credit for some moderation.

It may be said that we have lost sight of Nosology. Let it be recollected, that we have been led to this extraneous matter in defending ourselves, and that our objection has been principally to the mistaken attempts at reducing medicine to a greater simplicity. In a practical art of such importance, true simplicity is the establishment of aphorisms to direct us in the treatment of acute, and in the diagnosis of chronic, complaints. No one has a higher respect for Sauvages, the first of the *methodic* nosologists, for the care with which he has described diseases, given their history and remedies, and for the number of authorities he has collected with so much labour and judgment. In him, all these are so conspicuous, that his METHOD appears little more than the means of leading us from one object to another, and, as in the instance we have shown, of marking certain discriminations with greater accuracy. Such too was Cullen's wish, as appears by the quotation produced by Mr. Good; and, when Nosology is confined to these objects, we shall know how to estimate its worth. But, if diseases are ever to be classed like the more uniform productions of nature, it must be in a more advanced state of knowledge than we now possess; and we are still of opinion, that, in acute cases, the difficulty is the greatest, and the attempt has proved dangerous.

